# Duplex Study of the Nipple–Areola Complex Blood Supply in the Female Breast

**DOI:** 10.1093/asjof/ojae090

**Published:** 2024-10-24

**Authors:** Husam Hosny, Sahar Mansour, Nadeen M El-Essawy

## Abstract

**Background:**

Although breast blood supply comes from the internal mammary, lateral thoracic, and intercostal artery perforators, the nipple–areola complex (NAC) blood supply was usually depicted as a direct continuation of those vessels rather than a true description based on anatomical research. Studies focusing on NAC vascularity are few in number, done on a limited number of subject based on microdissection, computed tomography, or MRI. The results are inconsistent and may be perplexing. Hence, the need for studies on a large number of living subject is still warranted. Because duplex proved efficient at detecting perforators, we used it to assess NAC vascularity.

**Objectives:**

In vivo delineation of NAC blood supply by comparing this to the nipple-to-suprasternal notch (N-SN) distance and individual variation between both sides.

**Methods:**

Female subject presenting to the Breast Imaging Unit of the institute (229 subject; 458 breasts, BIRADS I and II) were assessed by duplex for the presence of significant NAC blood supply (≥1 mm), regarding their number, source vessel, perforator level, relation to N-SN distance, and similarity between both sides.

**Results:**

The third and fourth internal mammary artery (IMA) perforators accounted for 54.8% and 31.3%, respectively. The second IMA, lateral thoracic artery, and direct axillary branch were found in 9.2%, 3.3%, and 1.3%, respectively. The longer the N-SN distance is, the lower the supplying perforator. NAC vascularity was symmetric in 143 subject (62.4%) and asymmetric in 86 subject (37.5%).

**Conclusions:**

The third and fourth internal mammary perforators are the main source of NAC blood supply. Right and left sides’ asymmetry is not uncommon. Finally, the longer the N-SN distance is, the lower the perforator level is anticipated.

**Level of Evidence: 1 (Diagnostic):**

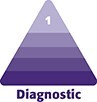

Many mammoplasty aesthetic and reconstructive techniques comprise nipple–areola complex (NAC) repositioning to a new position on top of a newly reconstructed breast mound. Thus, ensuring adequate blood supply to NAC is paramount in order to avoid the most devastating complication of partial or total NAC loss.

Although the breast and chest wall blood supply have been clearly described in the literature to come from the internal mammary artery (IMA), lateral thoracic artery (LTA), intercostal vessels, and thoracoacromial system,^1,2^ the NAC blood supply was depicted as radial continuations of those vessels toward the nipple rather than relying on studies that focus on NAC blood supply in specific. Moreover, the results of such studies are perplexing and sometimes contradictory.

Reported attempts at identifying NAC vascularity started in the 19th century, when Manchot^3^ described the NAC blood supply as coming from the IMA through 6 and 7 perforators, the LTA,^3-7^ intercostal space perforators, and a superficial thoracic artery; a branch from the axillary artery separate from the LTA. Since then, many cadaveric studies have been done, but their results varied in identifying the main source and number of perforators involved, and they did not compare both sides within the same individual. Moreover, their age and any premortem breast disease, if any, were not included. Marcus,^4^ Carr et al,^5^ van Deventer,^6^ and Anson et al^7^ recognized first to fourth or fifth IMA perforators to be the main source, with the second or third perforator being the predominant. But they differed in the order of perforator contribution and the role of LTA. On the other hand, O’Dey et al^8^ reported the LTA perforators to be the main source and only the second and fourth IMA perforators to share in the NAC blood supply. Würinger et al^9^ described a suspensory ligament above which the LTA and thoracoacromial perforators are the source vessels, whereas below it, the IMA and intercostal perforators give their contribution. Furthermore, le Roux et al,^10^ while studying preservation of the neurovascular supply in Hall-Findlay superomedial pedicle technique, demonstrated a single dominant vessel originating from the third or fourth internal mammary perforator that runs in a superficial plane, ranging ∼1 cm below the skin, heading toward the NAC.

Seitz et al^11^ proposed a new classification system for NAC vascularity, “NACSOMES,” dividing the breast into 5 zones in their in vivo MRI study of 52 breasts and reported the medial zone through IMA to be the main source. In addition, Wang et al^12^ compared NAC in hypertrophic vs normal breasts using MRI and reported a richer blood supply from the superomedial zone, followed by the superolateral and central zones.

In 1998, Blondeel et al^13^ reported using the Doppler flowmetry as an efficient tool in identifying reliable perforators for flap surgery and have used it in many limb and breast reduction and reconstruction surgeries.^14-16^ It allows identification of the exact anatomical features of the perforator, including its location, subcutaneous pathway, and point of entry to the deep fascia. In addition, it can also help detect the velocity and volume of flow inside the perforator, which guides the surgeons toward choosing the best available perforator-based flap.^14^

Hence, it was the idea of the first author to assess the NAC source of blood supply using the duplex on a large scale of healthy adult females to find out significant source vessel(s), revealing a possible correlation between the source vessel and the nipple-to-suprasternal notch (N-SN) distance and comparing both sides within the same individual (primary outcomes).

## METHODS

This is a prospective study, conducted at Cairo University Hospital in the time period between July 2020 and January 2022, following the rules and guidelines of the Research Ethics Committee of the Faculty of Medicine, Cairo University (REC N382024) with written consents obtained. Subject presenting to the breast imaging unit for routine screening or referred by their physicians were first assessed for findings suggesting abnormalities in breast parenchyma. Their age, complaints, previous breast surgeries, and upright N-SN distance were recorded.

Subject with suspected breast malignancy were then excluded from this study. Consequently, 229 healthy female subject out of 300 total subject were enrolled in the study, aiming to detect significant (>1 mm with good triphasic flow) arterial blood supply to their NAC and trace them back to their source vessel.

Imaging of the breast parenchyma was done initially using a Samsung-HS-60 ultrasound machine (Seoul, South Korea); after that, color duplex was applied using low-frequency pulsed Doppler waves and adjusted color gain.

The procedure was done in the supine position by moving the US probe around the whole perimeter of the NAC, and if any significant arterial perforator was detected, it was traced back to its main source vessel, as mentioned earlier (Video).

Data were then recorded through Microsoft Excel 2013 (Redmond, WA) and sent for statistical analysis using the statistical package for social science (SPSS) version 22 (SPSS, IBM, Armonk, NY). Simple descriptive statistics (arithmetic mean, standard deviation) were used for the summary of quantitative data, whereas frequencies were used for qualitative data. A bivariate relationship was displayed in cross tabulation, and comparison of proportions was performed using the χ^2^ test or Fisher exact test whenever appropriate. T-independent 1-way analysis of variance and post hook tests were used to compare normally distributed quantitative data. The level of significance was set at a probability (*P*-value) <.05.

## RESULTS

A total of 229 females (458 breasts, BIRADS I and II) were enrolled in this study. The mean age was 45 years ± 11 (range, 25-70 years). The mean N-SN on the right side was 38.55 cm (±8.63), whereas on the left side, it was 38.19 cm (±8.46; Table 1).

**Table 1.** Criteria of Studied Subject

	Mean	Minimum	Maximum	Standard deviation	Percentile 25	Percentile 75
Age	45	25	70	11	36	52
N-SN (R), cm	38.55	24	66	8.63	32	43
N-SN (L), cm	38.19	26	67	8.46	31	42

N-SN, nipple-to-suprasternal notch.

**Figure 1.**
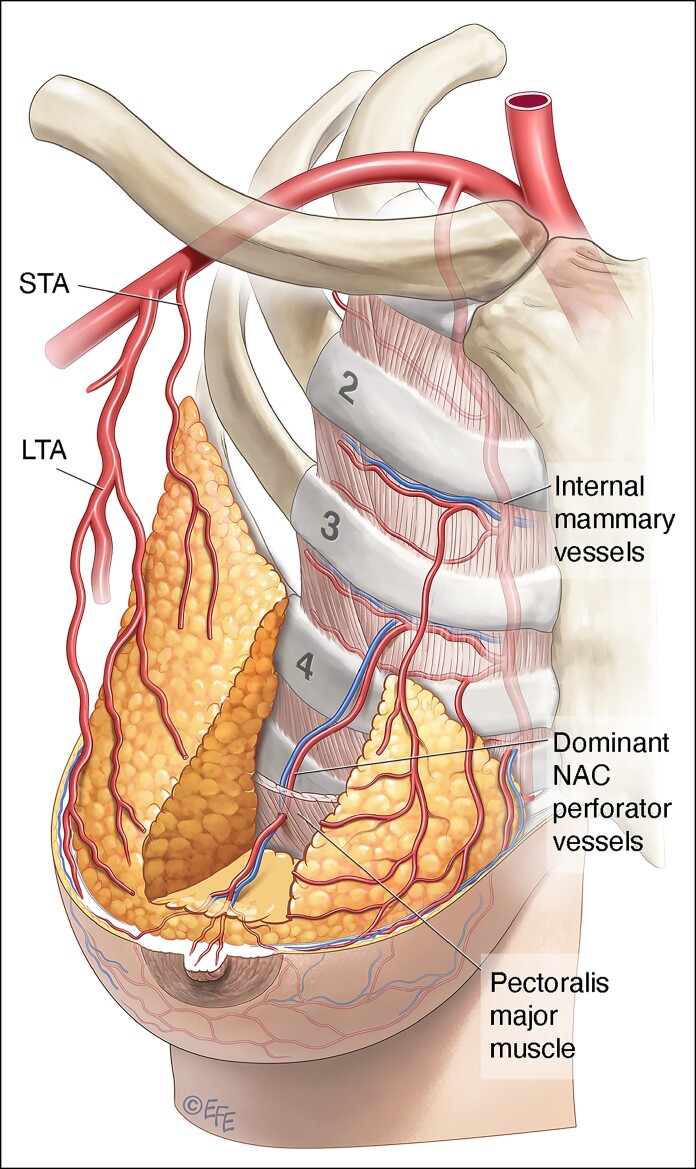
Depiction illustrating the course of a dominant perforator running in the superficial plane arising from an internal mammary perforator: (1) the sternum; (2) pectoralis major muscle; (3) internal mammary vessels; (4) the dominant nipple–areola complex perforator vessels.

In all subject, only 1 significant arterial vessel (≥1 mm in diameter) running in the superficial plane ∼1 cm from the skin could be found and traced from NAC to its original source vessel (Figure 1).

**Figure 2.**
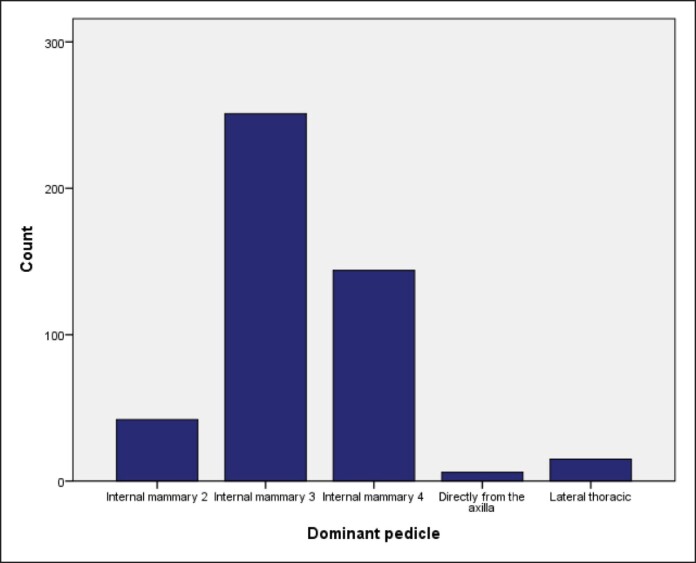
Bar chart showing the distribution of source vessels.

**Figure 3.**
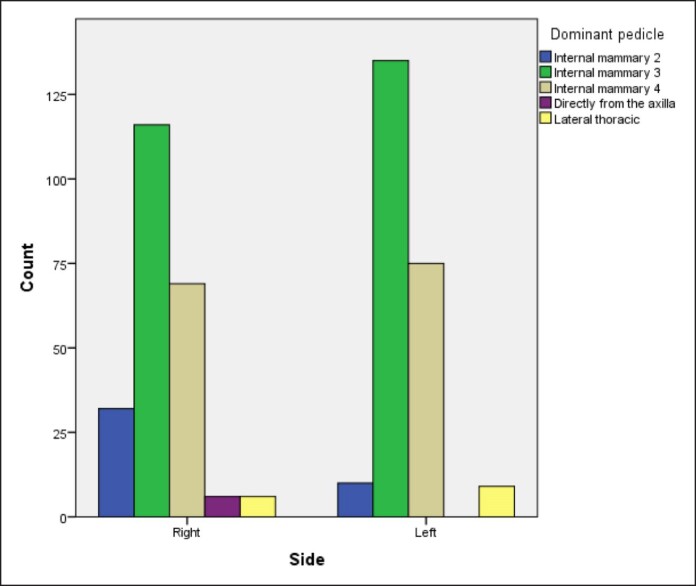
Bar chart showing the difference of distribution of source vessels between the right and left breasts.

In most cases, this vessel originated from the third (IM3) or fourth (IM4) IMA perforator in 251 (54.5%) and 144 breasts (31.4%), respectively (Table 2, Figure 2) and that also applied to both sides (Figure 3). IM2 was the main blood supply in 42 breasts (9.2%), whereas the LTA and direct axillary branch (DAB) were encountered in 15 (3.3%) and 6 breasts (1.3%), respectively (Table 2). Thus, collectively, internal mammary perforators account for 95.4% of the NAC blood supply (Table 2).

**Table 2.** Distribution of Dominant Pedicles and N-SN Between the Right and Left Breasts

	Side						*P*-value
	Right		Left		Total		
	*n*	%	*n*	%	*n*	%	
*N-SN (categories)*							.977
<30	33	14.4	36	15.7	69	15.1	
31-35	71	31.0	63	27.5	134	29.3	
36-40	50	21.8	46	20.1	96	21.0	
41-45	39	17.0	46	20.1	85	18.6	
46-50	13	5.7	16	7.0	29	6.3	
51-55	13	5.7	12	5.2	25	5.5	
56-60	3	1.3	3	1.3	6	1.3	
61-65	5	2.2%	6	2.6	11	2.4	
>65	2	0.9	1	0.4	3	0.7	
*Dominant pedicle*							.001
Internal mammary 2	32	14.0	10	4.4	42	9.2	
Internal mammary 3	116	50.7	135	59.0	251	54.8	
Internal mammary 4	69	30.1	75	32.8	144	31.4	
Directly from the axilla	6	2.6	0	0.0	6	1.3	
Lateral thoracic	6	2.6	9	3.9	15	3.3	

N-SN, nipple-to-suprasternal notch.

Further analysis categorizing subject according to the distance from N-SN revealed that most breasts measured between 31 and 45 cm (Table 2).

**Figure 4.**
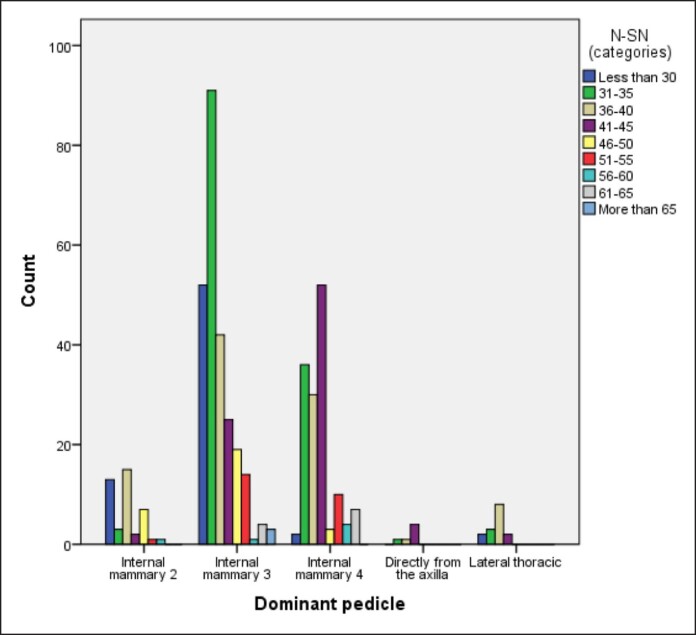
Bar chart showing relationship between different N-SN categories and the source vessel. N-SN, nipple-to-suprasternal notch.

We were also able to detect a direct correlation between N-SN and the major feeding perforator, where it was found that IM3 is the most prevalent NAC blood supply in 251 breasts (54%), especially with N-SN ≤ 40 cm (Table 3). Furthermore, IM4 was the second most prevalent, accounting for the blood supply of 144 breasts (31.4%), especially with long N-SN distances 41 to 45 cm as well as >55 cm (Table 3, Figure 4).

**Table 3.** Relationship Between N-SN and the Source Vessel

	Dominant pedicle												*P*-value
	IM2		IM3		IM4		DAB		Lateral thoracic		Total		
	*n*	%	*n*	%	*n*	%	*n*	%	*n*	%	*n*	%	
*N-SN (categories)*													<.001
<30	13	18.8	52	75.4	2	2.9	0	0.0	2	2.9	69	100.0	
31-35	3	2.2	91	67.9	36	26.9	1	0.7	3	2.2	134	100.0	
36-40	15	15.6	42	43.8	30	31.3	1	1.0	8	8.3	96	100.0	
41-45	2	2.4	25	29.4	52	61.2	4	4.7	2	2.4	85	100.0	
46-50	7	24.1	19	65.5	3	10.3	0	0.0	0	0.0	29	100.0	
51-55	1	4.0	14	56.0	10	40.0	0	0.0	0	0.0	25	100.0	
56-60	1	16.7	1	16.7	4	66.7	0	0.0	0	0.0	6	100.0	
61-65	0	0.0	4	36.4	7	63.6	0	0.0	0	0.0	11	100.0	
>65	0	0.0	3	100.0	0	0.0	0	0.0	0	0.0	3	100.0	
Total	42	9.2	251	54.8	144	31.4	6	1.3	15	3.3	458	100.0	

DAB, direct axillary branch; N-SN, nipple-to-suprasternal notch.

IM2 was not an uncommon source, as it supplied 42 breasts (9.2%), especially with shorter N-SN <30 cm; however, it can still be encountered in longer measurements (Table 3, Figure 4).

LTA and DAB were the least likely to supply the NAC, accounting for the blood supply in only 15 breasts (3.3%) and 6 breasts (1.3%), respectively, in different N-SN categories (Table 3, Figure 4).

**Figure 5.**
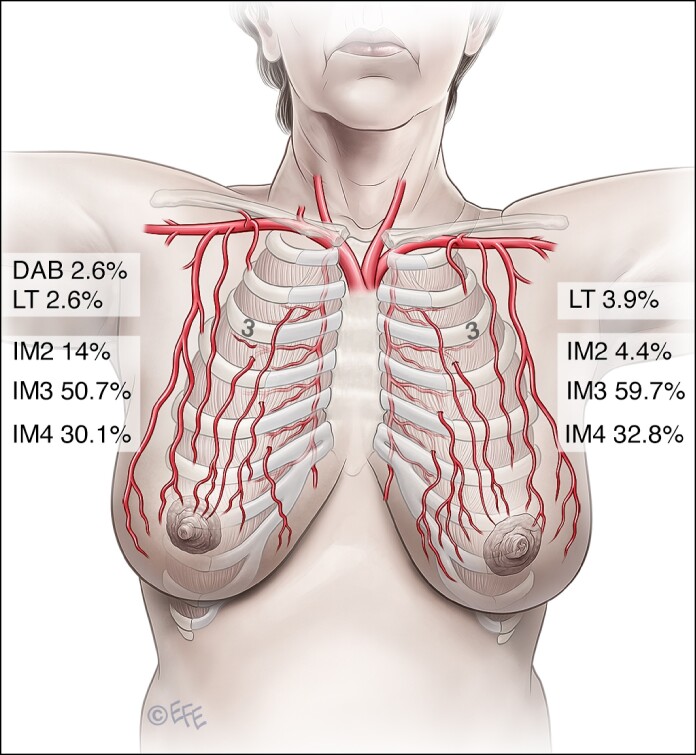
Distribution of the dominant perforator and its source vessels between the left and right sides.

Finally, comparing the NAC vascularity on the right vs the left sides in the same subject revealed that they were only symmetric (same source vessel) in 143 subject (62.4%), whereas in the remaining 86 subject (37.5%), the blood supply was asymmetric (different source vessels; Table 4, Figure 5).

**Table 4.** Distribution of Symmetric and Asymmetric Cases

	*n*	%	*P*-value
IM2-IM2	4	1.7	.036
IM3-IM3	85	37.1	<.001
IM4-IM4	49	21.4	<.001
LT-LT	5	2.2	<.001
DFA-DFA	0	0	—
Subtotal	143	62.44	
IM(R)-IM(L) collectively	213	93	<.001
IM2(R)-IM3(L)	25	10.9	.017
IM2(R)-IM4(L)	3	1.3	.002
IM2(R)-LT(L)	0	0	.617
IM2(R)-DFA(L)	—	—	—
IM3(R)-IM2(L)	6	2.6	.749
IM3(R)-IM4(L)	22	9.6	<.001
IM3(R)-LT(L)	3	1.3	.329
IM3(R)-DFA(L)	—	—	—
IM4(R)-IM2(L)	0	0	.035
IM4(R)-IM3(L)	19	8.3	<.001
IM4(R)-LT(L)	1	0.43	.284
IM4(R)-DFA(L)	—	—	—
LT(R)-IM2(L)	0	0	1.000
LT(R)-IM3(L)	0	0	.004
LT(R)-IM4(L)	1	0.43	.667
LT(R)-DFA(L)	—	—	—
DAB(R)-IM2(L)	0	0	1.000
DAB(R)-IM3(L)	5	2.18	.405
DAB(R)-IM4(L)	0	0	.181
DAB(R)-LT(L)	1	0.43	.216
Subtotal	86	37.55	

## DISCUSSION

Many breast oncoplastic, reconstructive, and aesthetic surgeries entail NAC repositioning to a new site on top of a newly formed breast mound. However, partial or total NAC loss because of vascular compromise is the most dreadful complication a plastic surgeon can ever meet. Thus, ensuring good NAC vascularity is paramount.

Although the chest wall and breast blood supply is thoroughly described in the literature, studies that focus on NAC vascularity are few in number, done on a limited number of cadavers, and have yielded inconsistent results. Moreover, Hyodoh et al,^17^ while comparing premortem and postmortem changes in 12 individuals using whole-body computed tomography (CT), found significant reductions (*P* < 0.05) on the long-axis diameter (79.2%-85%, mean 81.3%), the short-axis diameter (55.6%-80%, mean 68%), and the square of the radius of the aorta (48.5%-71.4%, mean 60.8%) compared with the antemortem measurements. Thus, the likelihood of cadaveric studies to match real-life situations might be questionable, and obtaining data from studies on large numbers of live individuals is still needed. In vivo studies are still scarce, mainly using CT angiography^18^ or MRI.^19^ Their main drawbacks are exposure to radiation and/or a high cost, and they were also done on a limited number of subject. On the other hand, duplex flowmetry has proven to be an efficient tool in identifying perforators and planning many flaps with reliable pedicles.^13^ Thus, using it to identify NAC vascularity may be justified and can be used to study large numbers of individuals with good accuracy, no radiation exposure risk, and cost-effectiveness.

In this work, 458 breasts (229 subject) were evaluated. This exceeds by far any previously published cadaveric or in vivo studies on NAC vascularity. Their age ranged from 25 to 70 years (mean 45 years), data not mentioned in the previous publications, representing the age at which many breast surgeries are performed for many aesthetic and reconstructive surgical procedures. Moreover, in this study, the source vessel is compared between both sides within the same individual and also correlated to the N-SN distance.

The IMA has been considered a major contributor to NAC blood supply^3-7^ through its perforators. However, the dominant perforator varies greatly among different studies.

In this study, the internal mammary perforators were found to be the main contributor (95.4%) to NAC blood supply (*P* = .001) through a single perforator, namely the IM3, IM4, and IM2 perforators in that order. This finding is in concordance with the study by le Roux et al,^10^ who also found a single dominant perforator supporting NAC vascularity. In our study, IM3 was the most prevalent source (54.8%) vessel both on the right (50.7%) and left (59%) sides (Table 2, Figures 2, 5).

IM4 comes in second among internal mammary perforators (total 31.4%, right side 30.1%, left side 32.8%). IM2 is the least likely to be the principal source of NAC blood supply (9.2%). Thus, the order of frequency differs from that described in the previous studies. However, we could not detect any significant source vessels above IM2 or below IM4 in our subject, contrary to Manchot^3^ and Anson et al^7^ who described perforators above IM2 and below IM4 as source vessels.

Regarding LTA, it was found as a main source in only 15 breasts (3.3%, *P* = .001; Table 2). However, anastomotic vessels between LTA and IMA could not be detected with duplex flowmetry and is beyond the capability of this technique, a limitation of this study.

In our work, we could find a separate branch coming directly from the axillary artery and heading toward the NAC in 6 breasts (1.3%, *P* *=* .001), referred to as DAB. This might be the same branch described by Manchot^3^ in 18,993 which he called the superficial thoracic artery, which is different from the highest thoracic (intercostal) artery described by O’Dey et al.^8^

Although anterior intercostal perforators are known for their contribution to the chest wall and breast blood supply, in this study, we could not find any significant intercostal perforators that reach NAC. This is in concordance with the results of many previous studies,^4,6,8,9^ reporting that the role of intercostal perforators in NAC blood supply is not yet obvious. Also, we could not identify significant perforators from the thoracoacromial axis reaching the NAC. The absence of significant perforators from intercostal and thoracoacromial vessels might explain the increased risk of partial or total NAC loss with long superior or inferior pedicles.

Correlating N-SN distance to source vessel revealed that IM3 is the main source of NAC blood supply in distances up to 40 cm (*P* < .001), whereas IM4 is the main source in long N-SN distances (41-45 and >55 cm, *P* < .001; Table 3, Figure 3). This observation may raise the question of whether the source vessel might change with increasing N-SN distance or whether it is inherent to each individual from birth and remains constant through her life.

Comparing the source vessel of the right and left breasts within the same individual revealed that in 143 subject (62.4%), both sides had the same source (*P* < .001), referred to as symmetric distribution (Table 4), whereas in 86 subject (37.6%), both sides had different sources, referred to as asymmetric cases; the distribution of different combinations and significance levels are summarized in Table 4. This pattern of asymmetry in NAC vascularity may explain vascular-related NAC complications that might happen in some mammoplasty cases in spite of exact preoperative planning, pedicle choice, and execution of the surgical procedure by the same surgeon. Thus, in those cases with an asymmetric NAC source vessel, there is always a potential risk of NAC ischemic complications, if the source vessel perforator is not included in NAC-bearing pedicles in mammoplasty procedures.

Also, in cases of severe ptosis and gigantomastia in which the NAC has to be displaced for a longer distance, including the perforator vessel in the long NAC-bearing pedicle should theoretically decrease ischemic-related complications, allow more volume resection, and avoid difficult insetting. However, there is an ongoing study in our institute about using perforator-based pedicles in gigantomastia to study the implication of including the NAC perforator within the pedicle in decreasing vascular-related complications. Hence, we assume that delineating the source vessel preoperatively and including it within the pedicle will turn it into an axial pattern dermoglandular flap—not confined to a strict length-to-width ratio, instead of being a random pattern dermoglandular flap carrying NAC which should better follow a length-to-width ratio of 1:1 to avoid ischemia-related complications.

Finally, we think that future studies of the clinical implications of the concept of perforator-based NAC-bearing pedicle are warranted to assess improved survival, especially of long N-SN distances, a limitation of this study.

## CONCLUSIONS

The internal mammary perforators are the main source of NAC blood supply, mainly IM3 and IM4, with less contribution from IM2, lateral thoracic, or DAB. Asymmetry between right and left sides is not uncommon. Finally, the longer the N-SN distance is, the lower the perforator level is anticipated.
